# Dysregulation of Synaptic and Developmental Transcriptomic/Proteomic Profiles upon Depletion of MUNC18-1

**DOI:** 10.1523/ENEURO.0186-22.2022

**Published:** 2022-11-09

**Authors:** Annemiek A. Van Berkel, Frank Koopmans, Miguel Angel Gonzalez-Lozano, Hanna C. A. Lammertse, Femke Feringa, Julien Bryois, Patrick F. Sullivan, August B. Smit, Ruud F. Toonen, Matthijs Verhage

**Affiliations:** 1 Department Functional Genomics, Center for Neurogenomics and Cognitive Research, Vrije Universiteit Amsterdam, Amsterdam 1081 HV, The Netherlands; 2 Department Molecular and Cellular Neurobiology, Center for Neurogenomics and Cognitive Research, Vrije Universiteit Amsterdam, Amsterdam 1081 HV, The Netherlands; 3 Functional Genomics, Department of Human Genetics, Center for Neurogenomics and Cognitive Research, Amsterdam UMC, Amsterdam 1081 HV, The Netherlands; 4 University of North Carolina Center for Psychiatric Genomics, University of North Carolina at Chapel Hill, Chapel Hill, NC 27599-7160; 5 Department of Medical Epidemiology and Biostatistics, Karolinska Institutet, Stockholm 171 77, Sweden

**Keywords:** MUNC18-1, neurodegeneration, neurodevelopment, proteomics, synapse, transcriptomics

## Abstract

Absence of presynaptic protein MUNC18-1 (gene: *Stxbp1*) leads to neuronal cell death at an immature stage before synapse formation. Here, we performed transcriptomic and proteomic profiling of immature *Stxbp1* knock-out (KO) cells to discover which cellular processes depend on MUNC18-1. Hippocampi of *Stxbp1* KO mice showed cell type-specific dysregulation of 2123 transcripts primarily related to synaptic transmission and immune response. To further investigate direct, neuron-specific effects of MUNC18-1 depletion, a proteomic screen was performed on murine neuronal cultures at two developmental timepoints before onset of neuron degeneration. 399 proteins were differentially expressed, which were primarily involved in synaptic function (especially synaptic vesicle exocytosis) and neuron development. We further show that many of the downregulated proteins on loss of MUNC18-1 are normally upregulated during this developmental stage. Thus, absence of MUNC18-1 extensively dysregulates the transcriptome and proteome, primarily affecting synaptic and developmental profiles. Lack of synaptic activity is unlikely to underlie these effects, as the changes were observed in immature neurons without functional synapses, and minimal overlap was found to activity-dependent proteins. We hypothesize that presence of MUNC18-1 is essential to advance neuron development, serving as a “checkpoint” for neurons to initiate cell death in its absence.

## Significance Statement

Presynaptic protein MUNC18-1 is essential for neuronal functioning. Pathogenic variants in its gene, *STXBP1*, are among the most common found in patients with developmental delay and epilepsy. To discern the pathogenesis in these patients, a thorough understanding of MUNC18-1’s function in neurons is required. Here, we show that loss of MUNC18-1 results in extensive dysregulation of synaptic and developmental proteins in immature neurons before synapse formation. Many of the downregulated proteins are normally upregulated during this developmental stage. This indicates that MUNC18-1 is a critical regulator of neuronal development, which could play an important role in the pathogenesis of *STXBP1* variant carriers.

## Introduction

The presynaptic protein MUNC18-1 (encoded by the gene *Stxbp1*) is implicated in SNARE-mediated fusion of synaptic and dense-core vesicles (Verhage et al., 2000; Puntman et al., 2021). Its absence not only arrests vesicle exocytosis, it also triggers extensive cell-autonomous neurodegeneration (Verhage et al., 2000; Heeroma et al., 2004). Remarkably, MUNC18-1’s role in neuronal viability is independent from its known function in vesicle exocytosis. Viability but not vesicle exocytosis is rescued on expression of noncognate paralogs (Santos et al., 2017; Puntman et al., 2021). Moreover, depletion of other presynaptic proteins essential for synaptic transmission, MUNC13 and VAMP2, does not result in degeneration (Schoch, 2001; Varoqueaux et al., 2002). Lastly, degeneration *in vitro* occurs before neurons have formed synapses and hence before synaptic transmission (Verhage et al., 2000). It remains to be elucidated why neuronal viability critically depends on MUNC18-1 expression.

Interestingly, neuronal cell death in *Stxbp1* null mutant [knock-out (KO)] brains follows a developmental pattern, starting at lower brain areas that mature first and gradually moves to higher brain areas that develop last (Verhage et al., 2000). A histochemical time series of *Stxbp1* KO brains showed that neurogenesis, overall organization, and early neuron differentiation are unaffected whereas at later time points [embryonic day (E)18] *Stxbp1* KO neurons fall behind in maturation (Verhage et al., 2000; Bouwman et al., 2004). E18 primary KO neurons can be maintained in culture, but are smaller in size, demonstrate reduced neurite outgrowth, and die after 3 d *in vitro* (DIV; Broeke et al., 2010; Santos et al., 2017). Conversely, primary cultures of E14 brains can be maintained in culture for 7 d (Santos et al., 2017). Together, these observations suggest that MUNC18-1 becomes critically involved in a process during neuron development which is distinct from its established role in synaptic transmission and is essential for neurons to survive.

To uncover which cellular processes become affected in the absence of MUNC18-1, transcriptomic and proteomic profiling was performed on *Stxbp1* KO mice shortly before the moment of cell death. We show that MUNC18-1 depletion strongly impacts the hippocampal transcriptome, and primarily affects transcripts related to synaptic transmission in neuronal cell types and immune response in non-neuronal cells. Mass spectrometry proteomics on neuron-specific primary cultures showed that loss of MUNC18-1 results in dysregulation of 399 proteins which are primarily involved in synaptic function and neuron development. High overlap was observed between downregulated proteins in *Stxbp1* KO neurons and proteins typically upregulated during this developmental stage. The proteomic changes showed minimal overlap to synaptic activity-dependent proteins. Together, these data demonstrate that MUNC18-1 regulates expression levels of an extensive set of synaptic and developmental proteins during neuronal development.

## Materials and Methods

### Animals

*Munc18-1* KO mice were generated as described previously (Verhage, 2000). Briefly, exons 2–6 were replaced with a neomycin resistance gene by homologous recombination, resulting in complete depletion of MUNC18-1 expression. Since depletion of MUNC18-1 is lethal on birth, KO mice were generated by crossing heterozygous mice. On E18 of pregnancy, mice were killed, and pups of either sex were obtained by caesarean section. Animals were housed and bred according to Institutional and Dutch governmental guidelines.

### RNA isolation

Hippocampi from E18 wild-type (WT) and *Stxbp1* KO littermates were collected and snap-frozen in liquid nitrogen. Tissue was homogenized on ice. RNA isolation was performed using TRIzol and RNeasy Micro kit (QIAGEN). RNA sample quality was assessed on a NanoDrop 2000 spectrophotometer (ThermoScientific).

### Neuronal cultures

Cortices were obtained from littermate-matched E18 wild-type and *Munc18-1* KO embryos and collected in HBSS (Sigma) containing 7 mm HEPES (Invitrogen). Tissue was incubated in Hanks’-HEPES with 0.25% trypsin (Invitrogen) for 20 min at 37°C. After three washes, cortices were triturated with fire polished Pasteur pipettes and neurons were counted in a Fuchs-Rosenthal chamber. Neurons were plated on 35-mm poly-L-ornithine/laminin-coated wells in prewarmed Neurobasal (Invitrogen) supplemented with 2% B27 (Invitrogen), 1.8% HEPES, 0.25% Glutamax (Invitrogen) and 0.1% Pen/Strep (Invitrogen). Cells were plated at a density of 800,000/well (WT) or 1,000,000/well (KO).

### RNA sequencing

Sequencing library preparation was performed using TruSeq stranded mRNA library preparation kit with poly A selection (Illumina Inc.). Cluster generation and paired-end sequencing was performed 125 cycles in one lane by Illumina HiSeq system, executed by SNP&SEQ Technology Platform at Uppsala Biomedical Centre. Base calls were converted to fastq format. Trimmomatic command was used to remove TruSeq3 adaptors. Quality control was performed using multiQC (see: multiqc_report_afterTrimmingAdaptors.html for QC report). Trimmed reads were mapped to mm10 (mouse) reference genome and quantified using Salmon. Differential expression analysis was performed using DESeq2 (R package). An FDR adjusted threshold of 0.005 was used to discriminate significantly regulated proteins.

Samples were clustered using hierarchical dendrogram and principal component analysis (PCA; Extended Data Fig. 1-2). Whereas most samples clustered according to genotype, two samples (WT3 and KO5) extremely deviated from other samples and these outliers were excluded.

**Figure 1.**
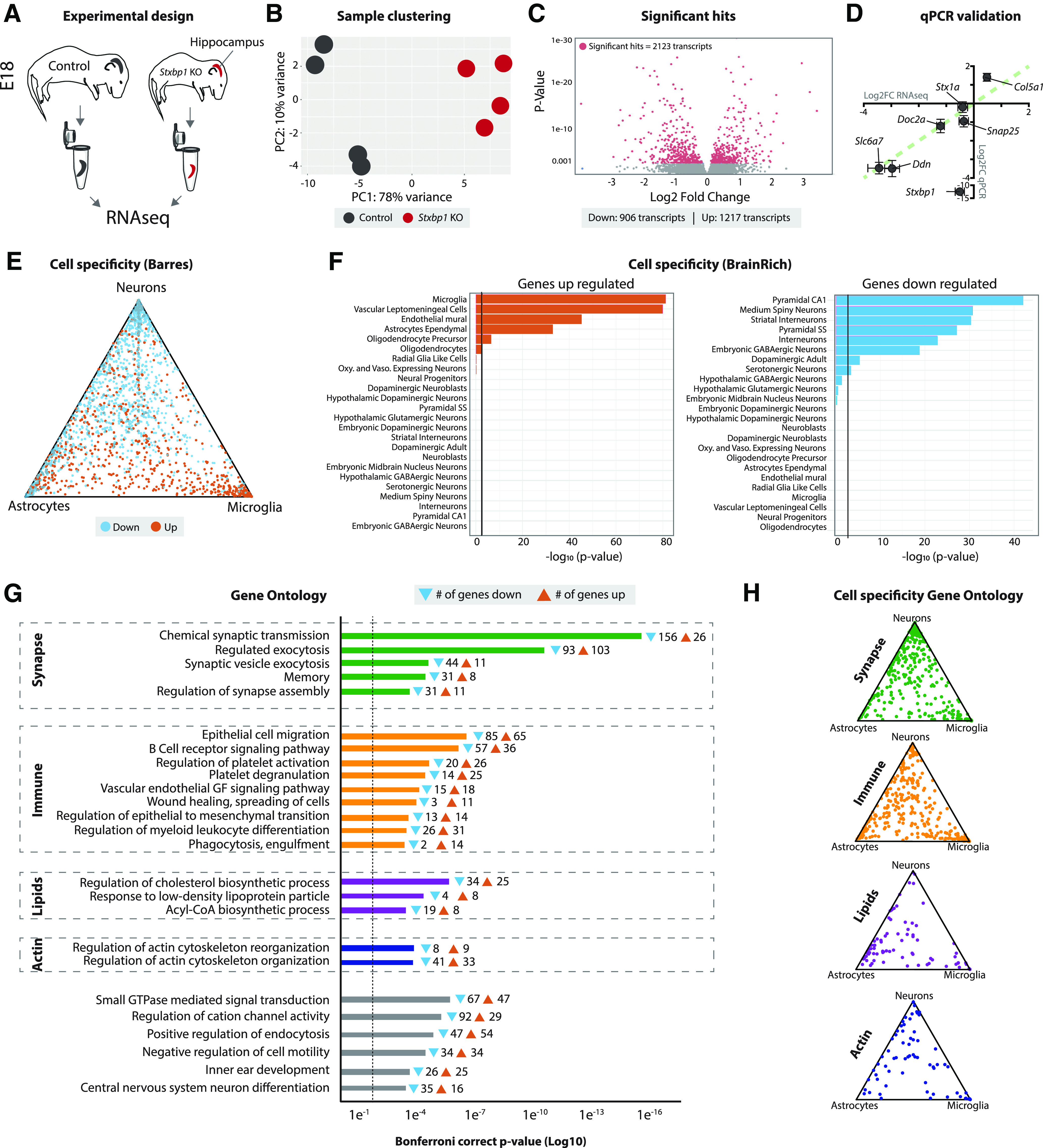
Cell type-specific transcripts related to synapse function, immune system, lipid metabolism, and actin organization are changed in *Stxbp1* KO hippocampi. ***A***, Cartoon of experimental design. E18 hippocampi were dissected from WT and *Stxbp1* KO brains, RNA was isolated, and RNAseq was performed. Typical examples of WT and KO E18 hippocampi are shown in Extended Data Figure 1-1. ***B***, PCA of transcript abundance levels showing PC1 (78% variance explained) and PC2 (10% variance explained) of control and *Stxbp1* KO samples. *N* = 4 independent replicates. Two outliers were excluded from analysis, see Extended Data Figure 1-2. ***C***, Volcano plot showing 2123 transcripts (906 downregulated, 1217 upregulated) significantly dysregulated in *Stxbp1* KO hippocampi from controls. ***D***, Several significant transcripts were validated using qPCR. Shown are Log2 Fold changes of transcripts using RNAseq (*x*-axis) and qPCR (*y*-axis). Green dotted line indicates correlation of 1. *N* = 6 independent replicates. Effect sizes were highly comparable for the included transcripts, except for *Stxbp1*. This is explained by the fact that despite the deletion of exons 2–6 in *Stxbp1*, a (nonfunctional) transcript is transcribed that is not detected by the qPCR primers (targeted within the deleted region). qPCR primers can be found in Extended Data Table 1-1. ***E***, Triangle plot showing cell specificity using the Barres RNAseq database. Downregulated transcripts are depicted in blue, upregulated transcripts in orange. ***F***, Bar graphs showing cell specificity using BrainRich. Upregulated transcripts are shown left, downregulated transcripts right. ***G***, GO enrichment analysis of the significant hits. Shown are the Bonferroni correct *p*-values and the number of transcripts associated with every GO term. ***H***, Triangle plots showing cell specificity of transcripts associated with GO term groups using the Barres RNAseq database.

**Figure 2.**
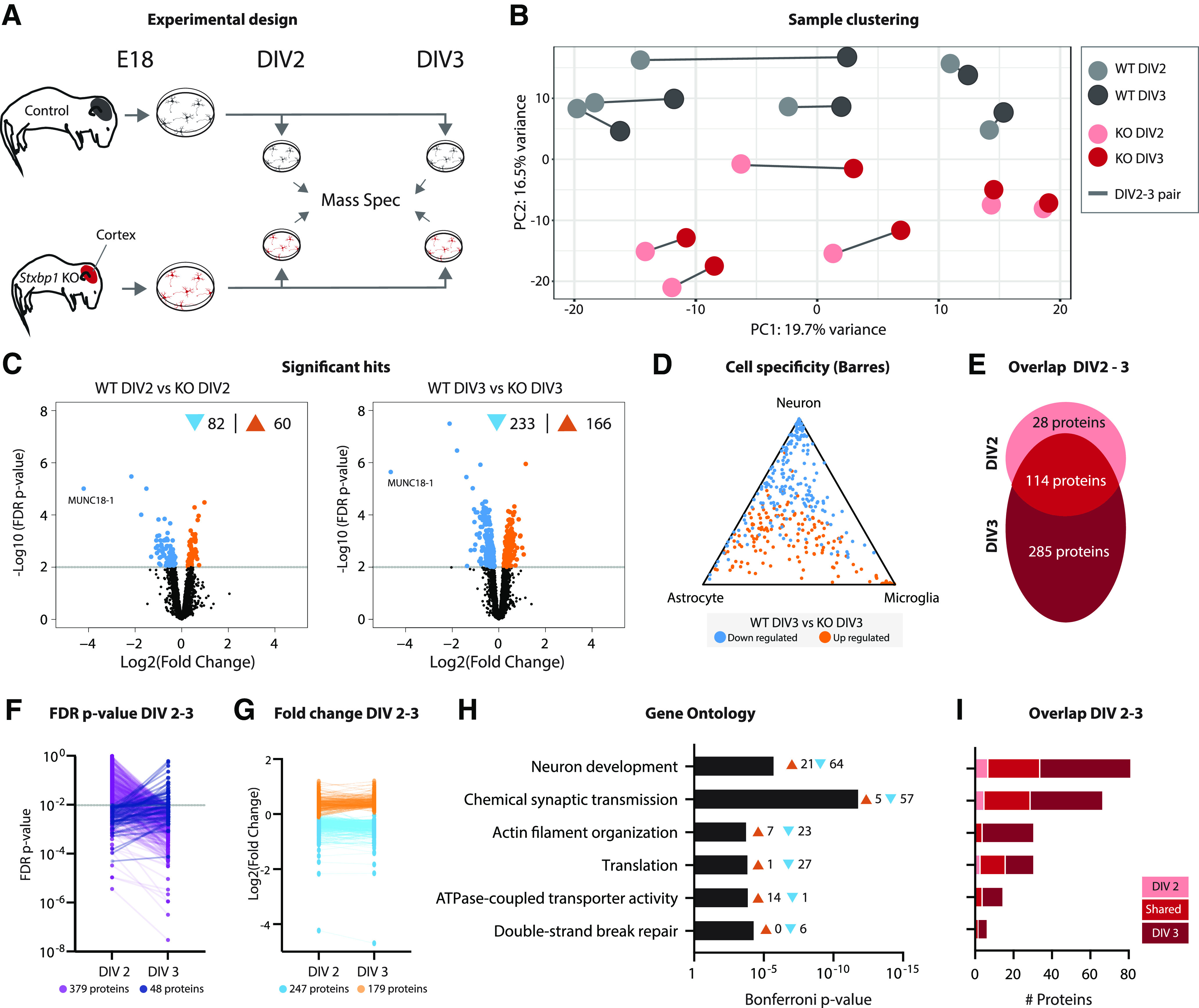
Proteins related to synapse function and neuron development are most severely affected in primary *Stxbp1* KO neurons. ***A***, Cartoon of experimental design. Primary neuronal cultures were generated from E18 WT and *Stxbp1* KO cortices. Neurons were harvested at DIV2 and DIV3 and analyzed using mass spectrometry. Quality control measures are shown in Extended Data Figure 2-1. ***B***, PCA of peptide abundance levels showing PC1 (19.7% variance explained) and PC2 (16.5% variance explained). *N* = 6 independent replicates. ***C***, Volcano plots showing significantly dysregulated proteins in *Stxbp1* KO neurons at DIV2 (142 proteins, 82 downregulated, 60 upregulated) and DIV3 (399 proteins, 233 downregulated, 166 upregulated). Volcano plots of regulated proteins between DIV2 and DIV3 are shown in Extended Data Figure 2-2. ***D***, Triangle plot showing cell specificity using the Barres RNAseq database. Downregulated transcripts are depicted in blue, upregulated transcripts in orange. ***E***, Overlap of significant proteins between DIV2 and DIV3. A total of 28 proteins were only significant at DIV2, 114 proteins significant at DIV2 and DIV3, and 285 only significant at DIV3. ***F***, FDR *p*-values at DIV2 and DIV3. For 379 proteins, *p*-values increased at DIV3, for 48 proteins *p*-values decreased. ***G***, Log2 fold changes at DIV2 and DIV3. Downregulated proteins are depicted in blue, upregulated in orange. ***H***, GO enrichment analysis of the significant hits. Shown are the Bonferroni correct *p*-values and the number of transcripts associated with every GO term. The proteins in significant GO terms can be found in Extended Data Table 2-1. ***I***, Overlap of proteins involved in enriched GO terms between DIV2 and DIV3.

10.1523/ENEURO.0186-22.2022.f1-2 Extended Data Figure 1-2 RNAseq outlier exclusion. A, Dendrogram of WT and KO samples. WT3 and KO5 separated from other samples. B, PCA of transcript abundance levels showing PC1 (51% variance explained) and PC2 (25% variance explained) of control and Stxbp1 KO samples. N = 5 independent replicates. WT and KO showed clear clusters, except for WT3 and KO5. Download Figure 1-2, EPS file.

### qPCR

A total of 500 ng RNA was reversed transcribed into cDNA using sensiFAST cDNA Synthesis kit (Bioline) according to manufacturer’s instructions. cDNA was quantified using SensiFAST SYBR No-ROX (Bioline) in a LightCycler 480 (Roche Life Sciences), using 10uM primers shown in Extended Data Table 1-1. The following program was used: 5-min incubation at 95°, 4.8C/s ramp rate, followed by 50 cycles of 10 s 95° (4.8 C/s), 20 s 60° (2.4 C/s), 1 s 72° (4.8 C/s). Primers showed clean melting curves. Cp values were determined using the second derivative maximum method. Samples were quantified in duplicates and average values were used. cDNA levels were normalized to 18S and EEF.

### Mass spectrometry-based proteomics

At 2 and 3 DIV, neuronal cultures were placed on ice and washed two times with ice-cold PBS. Next, 500ul PBS supplemented with protease inhibitor (PI) solution was added to each well and cells were collected by gentle scraping. Samples were centrifuged for 5 min at 3000 × *g* at 4°C, after which the supernatant was removed. The pellet was resuspended in 20-μl loading buffer (4% SDS, 100 mm Tris, pH 6.8, 0.04% bromophenol blue, 200 mm DTT, 20% glycerol, and PI in PBS). Samples were snap frozen and stored at −80°C until further processed. An SDS-PAGE LC‐MS/MS approach was used for protein identification as described previously (Gonzalez-Lozano and Koopmans, 2019). SWATH data were searched against a spectral library (peptides and proteins identified from DDA data by MaxQuant) of DIV2 and DIV3 neurons, using Spectronaut 13.7 (Bruderer et al., 2015) with default settings. The resulting abundance values and qualitative scores for each peptide in the spectral library were exported for further analysis.

R language was used for statistical computation. Only peptides present in WT and KO samples and quantified with high confidence (i.e., a q-value ≤ 10^−3^ over all samples in either group, allowing for one outlier within each condition) were included. Spectronaut normalized peak area was used to compute protein abundances, which were Loess normalized using the ‘normalizeCycleLoess’ function from limma R package (Rouillard et al., 2016). The ‘eBayes’ and ‘topTable’ functions from limma R package were implemented to perform empirical Bayes moderated *t*-statistics with multiple testing correction by FDR on log-transformed protein abundances. An FDR adjusted threshold of 0.01 was used to discriminate significantly regulated proteins.

### Bioinformatics

For all Gene Ontology (GO) and overlap analyses, the first gene name was used in case peptides were mapped to multiple genes. Fifteen significant hits shared the first gene name with (at least) one other hit. As a result, the total number of genes used for downstream analysis is lower (384 vs 399). Cytoscape plug-in ClueGO (Bindea et al., 2009) was used to perform GO analysis on RNAseq and proteomic data, using the Biological Process GO database updated on February 10, 2021. ClueGO analyses were performed including the following settings: Biological Process, GO term grouping, GO tree interval was set 6–8, GO terms consisting of at least five genes and min. 5% of the term. All detected transcripts/proteins were used as background. The GO fusion option was enabled, fusing GO terms that overlapped >50% of their significant genes. GO terms were grouped according to κ scores, and named after the most significant GO term. In Figures 1 and 2, only these grouped terms are shown. Cell type specificity was assessed using the Barres and BrainRich databases (Zhang et al., 2014; Skene et al., 2018). Barres cell specificity was visualized in Rstudio using the triangle.plot function in *ade4* package, using transcript levels of isolated neuronal, astroglial and microglial cell populations (Zhang et al., 2014). Functional annotation of synaptic proteins was done in the SynGO portal (https://syngoportal.org; Koopmans et al., 2019). Sunburst plot was made visualizing SynGO term enrichment of biological processes. MUNC18-1 interactors were identified using the STING database. Interactors were included that showed high confidence (>0.9), derived from experimental data and/or databases.

10.1523/ENEURO.0186-22.2022.f1-1 Extended Data Figure 1-1 Cleaved-Caspase 3 reactivity in E18 hippocampi of WT and Stxbp1 KO mice. A, Typical examples of E18 hippocampal slices from WT and Stxbp1 KO mice. Download Figure 1-1, EPS file.

10.1523/ENEURO.0186-22.2022.tab1-1 Extended Data Table 1-1 qPCR primers. Download Table 1-1, DOCX file.

10.1523/ENEURO.0186-22.2022.f2-1 Extended Data Figure 2-1 QC mass spectrometry proteomics. A, Q-values for peptides detected in at least one sample. B, CoV values for detected peptides for every experimental group. C, Dendrogram of WT and KO samples in Euclidean distances. Cluster confidence is shown in red. Download Figure 2-1, EPS file.

10.1523/ENEURO.0186-22.2022.f2-2 Extended Data Figure 2-2 Significant proteins between DIV2 and DIV3 in WT and KO. A, Volcano plots showing significantly dysregulated proteins between DIV2 and DIV3. For WT cultures, one protein was significantly upregulated at DIV3. For KO cultures, 24 proteins were significantly upregulated at DIV3. Download Figure 2-2, EPS file.

## Results

### *Stxbp1* KO hippocampal tissue shows cell type-specific dysregulation of transcripts related to synapse function, immune system, lipid metabolism, and actin organization

To profile transcriptional effects of *Stxbp1* depletion, bulk RNA sequencing was performed on E18 hippocampal tissue from WT and *Stxbp1* KO littermates (Fig. 1*A*). In contrast to lower brain areas, the hippocampus is still intact at E18 showing comparable cell density to WT, yet the first cells start to show markers of the apoptotic cell death pathway (Extended Data Fig. 1-1; Verhage et al., 2000; Bouwman et al., 2004). Sequencing detected 18 445 transcripts. Principal component analysis (PCA) showed a clear separation between genotypes (Fig. 1*B*). 11.5% transcripts were differentially regulated were significantly dysregulated in *Stxbp1* KO. A total of 906 transcripts were significantly downregulated in the *Stxbp1* KO, whereas 1217 transcripts were upregulated (Fig. 1*C*). qPCR analysis validated effect directionality and magnitude of several selected candidates (Fig. 1*D*). Together, depletion of *Stxbp1* results in a robust transcriptomic response in the E18 hippocampus.

To identify cell types where the differentially regulated genes are most likely expressed, two complementary cell-specific databases were used (Fig. 1*E*,*F*). First, we mapped all differentially regulated genes against the Barres RNASeq cell-type expression database (Zhang et al., 2014). Upregulated transcripts were mainly associated with microglia, whereas downregulated transcripts were associated with neurons (Fig. 1*E*). Second, this pattern was confirmed by comparison to expression patterns of single-cell RNAseq (Skene et al., 2018). Here, upregulated transcripts were enriched for several glial and vascular cell types. Consistently, downregulated transcripts were primarily associated to neuronal cell types (Fig. 1*F*). Hence, effect directionality on *Stxbp1* inactivation differs between cell types.

Gene Ontology (GO) analysis was performed to uncover enriched functional gene groups (Fig. 1*G*). The most significant group of GO terms were related to synapse function, encompassing a total of 213 genes. In addition, many genes were associated with GO terms in immune response (350 genes), lipid metabolism (92 genes), and actin organization (77 genes). Cell specificity analysis for the different GO groups showed that synaptic genes were primarily expressed in neurons, as expected, whereas immune and lipid genes showed a higher specificity toward glial cells. No cell specificity class was found for actin organization (Fig. 1*H*). Taken together, depletion of MUNC18-1 has differential effects on cell types. Transcripts associated with neurons were primarily downregulated, and associated with synapse function. In contrast, transcripts associated with glial cells were generally upregulated, and associated with immune response and lipid metabolism.

Previously, micro-array analysis on E18 *Stxbp1* KO and WT cortices identified 5586 transcripts, of which 9% were differently expressed (Bouwman et al., 2006). Most significant GO terms in that study overlap with the present *Stxbp1* KO dataset, including synaptic transmission, steroid metabolism, transmission of nerve impulse and cell-cell signaling. Thus, the RNAseq data confirms earlier microarray results on *Stxbp1* KO brains yet covers 3.3 times more transcripts.

### Proteome changes in *Stxbp1* KO neuron cultures relate to synapse function and neuron development

The presence of and crosstalk between different brain cell types in the bulk RNA sequencing results complicates interpretation of direct, neuron-intrinsic effects of MUNC18-1 depletion. In order to study neuron-specific protein regulation, mass spectrometry proteomics was performed on neuronal cultures from WT and *Stxbp1* KO E18 brains (Fig. 2*A*). Additional advantages of this approach include the developmental resynchronization of neurons allowing analysis at the same developmental stage (Dotti et al., 1988). To examine proteomic changes around the moment when neurons become dependent on MUNC18-1 for neuronal viability (Santos et al., 2017), two time points (DIV2 and DIV3) were selected for proteomic analysis.

In total, 3100 unique proteins were detected. PCA showed that samples segregated based on genotype, but not on time point (Fig. 2*B*). Samples derived from the same primary culture generally clustered together. At DIV2, 5% of the proteins were dysregulated in *Stxbp1* KO neurons, of which 82 were downregulated and 60 upregulated (Fig. 2*C*). The number of dysregulated proteins increased to 399 (13%) at DIV3; 233 downregulated and 166 upregulated. Compared with cell type transcript levels, proteins were generally either neuron-specific or nonspecific for any cell type (Fig. 2*D*). The vast majority (80%) of differentially expressed proteins at DIV2 were also dysregulated at DIV3 (Fig. 2*E*). However, at DIV3 many more unique proteins (285) were dysregulated. Indeed, 379 (89%) of all significant proteins became more significant at DIV3, whereas 48 (11%) proteins were less significant at DIV3 compared with DIV2 (Fig. 2*F*). Log2 fold changes of dysregulated proteins did not profoundly differ between the two time points (Fig. 2*G*), suggesting that the increase in significance is likely because of lower variation at DIV 3, as shown by the coefficient of variation (Extended Data Fig. 2-1). GO analysis revealed that biological processes related to neuron development and synaptic transmission were most prominently affected in KO neurons (Fig. 2*H*; Extended Data Table 2-1). In addition, proteins related to actin organization, translation, ATPase transporter activity and DNA break repair were also significantly dysregulated. None of the biological processes was unique for either time point, yet many proteins within these biological processes were only significant at DIV3 (Fig. 2*I*). In contrast to the bulk RNAseq results, no biological processes related to immune response or lipid metabolism were found, indicating that non-neuronal cell types most likely contributed to these processes in the bulk results. In addition to genotype effects, we also investigated protein level changes between DIV2 and DIV3 within WT or KO neurons (Extended Data Fig. 2-2). In WT neurons, the transition from DIV2 to DIV3 resulted in upregulated of one protein (MFGE8). KO neurons showed upregulation of 24 proteins. GO analysis did not reveal any specific biological process being regulated between DIV2 and DIV3. In sum, *Stxbp1* KO neurons show extensive remodeling of their proteome compared with WT neurons, which primarily affects proteins related to neuron development and synaptic function. Between DIV2 and DIV3, an increasing number of proteins involved in these processes become significantly dysregulated.

### Limited regulation of proteins involved in known cell death pathways in *Stxbp1* KO neurons

**Figure 3.**
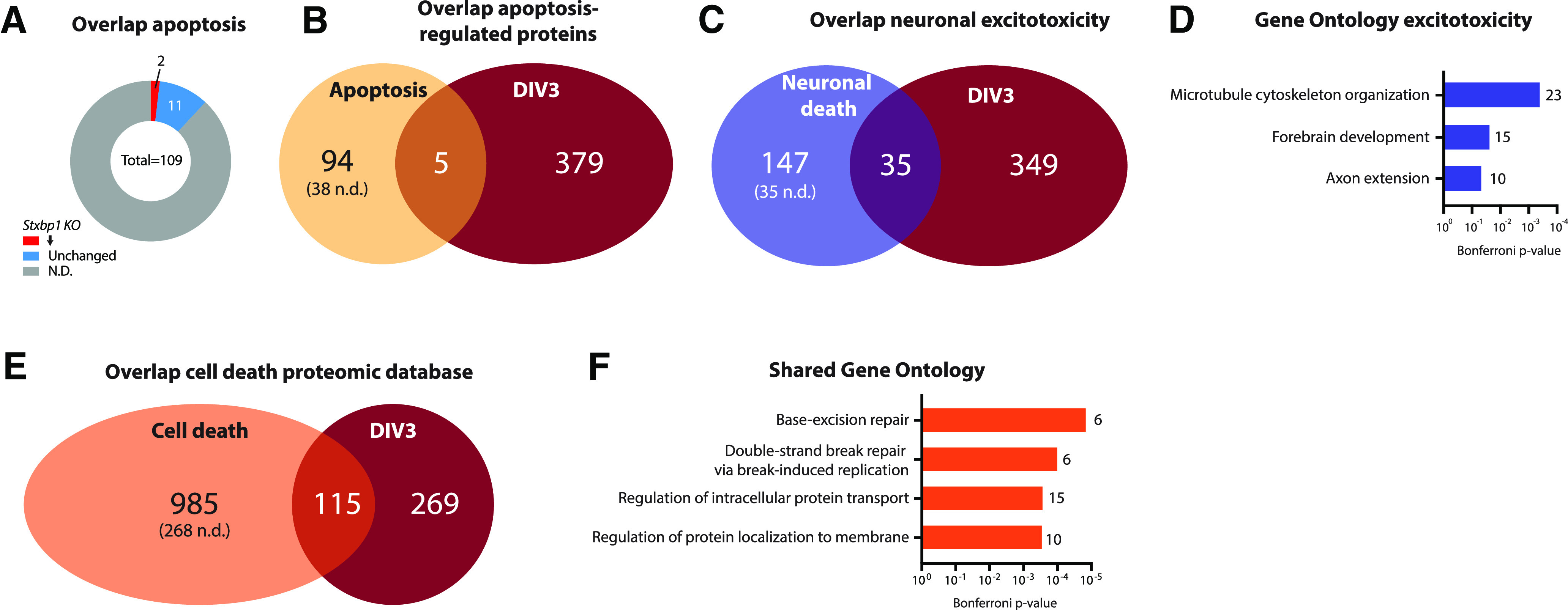
Dysregulated proteins in *Stxbp1* KO neurons at DIV3 show minimal overlap with cell death. ***A***, Overlap of significant proteins at DIV3 and proteins significantly regulated during apoptosis (Arntzen and Thiede, 2012). A total of 94 proteins were unique for apoptosis (38 not detected in *Stxbp1* KO dataset), five proteins shared between the two datasets, and 379 unique for *Stxbp1* KO neurons. ***B***, Overlap of significant proteins at DIV3 and proteins annotated to apoptosis in the KEGG pathway database. Of the 109 proteins annotated to apoptosis, 13 were detected in *Stxbp1* KO neurons. Two proteins were significantly downregulated. ***C***, Overlap of significant proteins at DIV3 and dysregulated proteins on excitotoxicity (Hoque et al., 2019). A total of 147 proteins were unique for excitotoxicity neuronal cell death (35 not detected in *Stxbp1* KO dataset), 35 proteins shared between the two datasets, and 349 unique for *Stxbp1* KO neurons. As for this analysis, only first gene names are used, resulting in 15 duplicates, the total number of significant *Stxbp1* KO proteins is lower (384 vs 399 in Fig. 2*C*). ***D***, GO enrichment analysis of significant dysregulated proteins on excitotoxicity. Shown are the Bonferroni correct *p*-values and number of proteins associated with each GO term. ***E***, Overlap of significant proteins at DIV3 and proteins significantly regulated during cell death (Arntzen and Thiede, 2012). A total of 985 proteins were unique for cell death (268 not detected in *Stxbp1* KO dataset), 115 proteins shared between the two datasets, and 269 unique for *Stxbp1* KO neurons. ***F***, GO enrichment analysis of significant dysregulated proteins on excitotoxicity. Shown are the Bonferroni correct *p*-values and number of proteins associated with each GO term. Detailed information on the overlap can be found in Extended Data Table 3-1.

Between DIV2 and DIV3 *Stxbp1* KO neurons show extensive cell death, ultimately involving, but not driven by, apoptosis (Verhage et al., 2000; Law et al., 2016; Santos et al., 2017). However, GO analysis of the proteomics dataset did not reveal evident cell death-related biological processes. To further characterize overlap with proteins typically regulated during cell death, proteins significantly regulated in *Stxbp1* KO neurons were compared with lists of proteins shown to be affected during different types of cell death. First, we focused on apoptosis-specific proteomic regulation in *Stxbp1* KO neurons. Minimal overlap was observed between the *Stxbp1* KO dataset and apoptosis genes in the KEGG pathway database as well as to apoptosis-regulated proteins identified in a database of published proteomic studies (Fig. 3*A*,*B*; Arntzen and Thiede, 2012). Together, we found no indication for prominent apoptosis-dependent protein regulation in *Stxbp1* KO neurons.

Next, regulated proteins were compared with proteins regulated during neuronal excitotoxicity (Hoque et al., 2019). The two datasets showed minimal overlap, with 91% of the proteins significant in *Stxbp1* KO neurons not regulated during excitotoxicity (Fig. 3*C*; Extended Data Table 3-1). None of the GO terms affected during excitotoxicity were shared with affected GO terms in *Stxbp1* KO neurons (Fig. 3*D*, compare to Fig. 2*H*). Thus, limited overlap is found between proteins regulated on excitotoxicity and KO of *Stxbp1*. The lack of overlap in synaptic transmission and neuron development GO terms indicates that these biological processes are not generally affected in neuronal cell death.

To further investigate overlap with other cell-death pathways, the *Stxbp1* KO dataset was compared with 1100 proteins associated with any type of cell death in (at least) two independent research studies, as assembled by Arntzen and Thiede (2012). A total of 115 proteins showed overlap to *Stxbp1* KO regulated proteins (Fig. 3*E*; Extended Data Table 3-1). GO analysis of these shared proteins did not reveal GO terms specific for any cell death pathway, but did show GO processes related to DNA repair and protein localization (Fig. 3*F*). Taken together, proteomic changes in *Stxbp1* KO neurons very limited overlap with apoptosis-induced changes, but show some overlap with general cell-death protein regulation. Further investigation of these shared regulated proteins could provide new insights on cell death pathways involved in *Stxbp1* KO neurons.

### The synaptic proteome is severely affected on depletion of MUNC18-1

**Figure 4.**
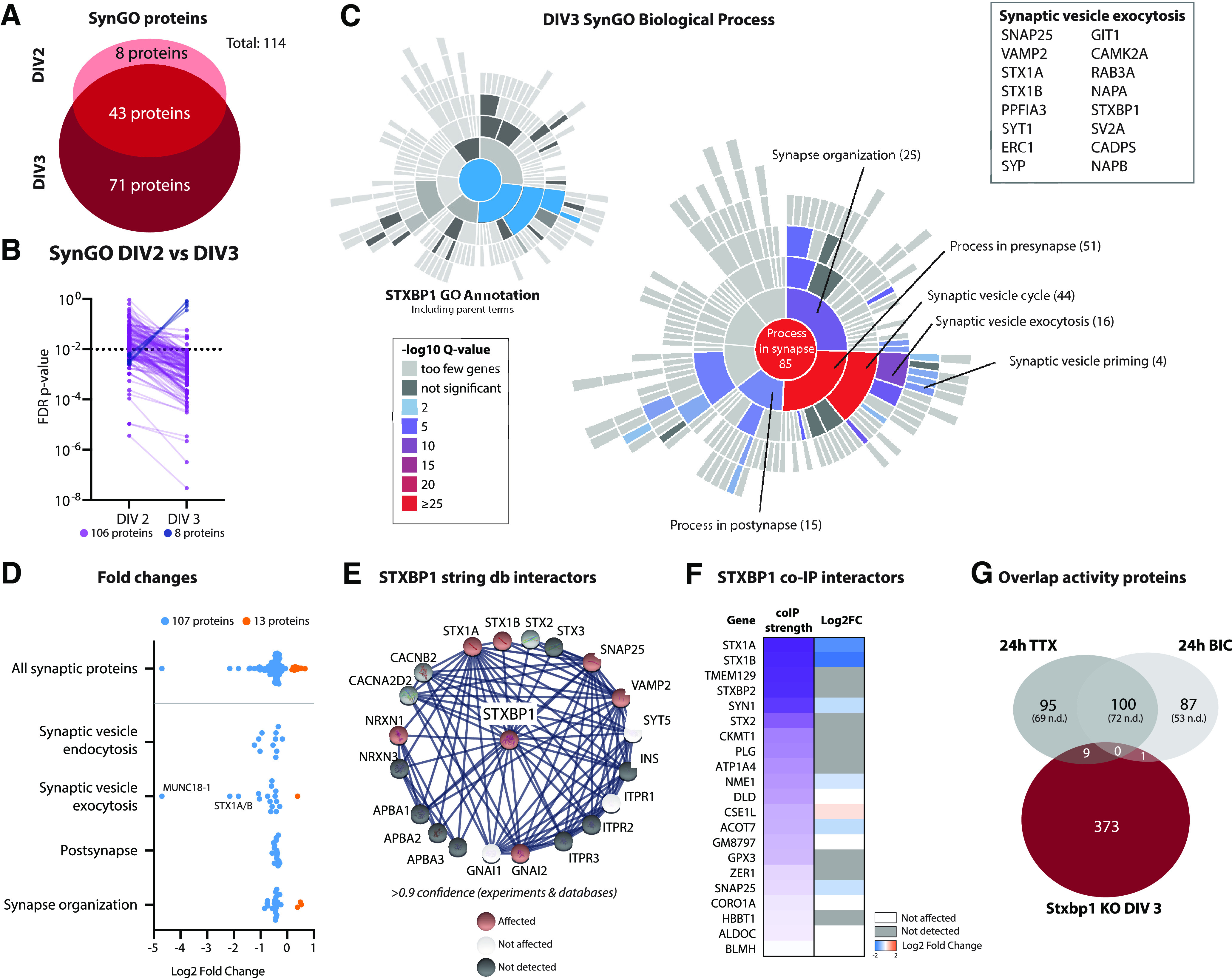
Depletion of MUNC18-1 leads to downregulation of 114 synaptic proteins enriched in presynaptic vesicle release. ***A***, A total of 114 of the significant proteins in *Stxbp1* KO neurons were annotated in SynGO. Eight synaptic proteins were only significant at DIV2, 43 synaptic proteins significant at DIV2 and DIV3, and 63 synaptic proteins only significant at DIV3. Overlap to the RNAseq SynGO genes is shown in Extended Data Figure 4-1. ***B***, FDR *p*-values of significant synaptic proteins at DIV2 and DIV3. For 106 synaptic proteins *p*-values increased at DIV3, for eight synaptic proteins *p*-values decreased. ***C***, A total of 85 synaptic proteins were categorized in SynGO biological processes. Shown is a sunburst plot with color-coded enrichment significance of every GO term. Left, SynGO annotation of MUNC18-1 in blue (including its parent terms). Right, Significant proteins associated with synaptic vesicle exocytosis. The SynGO protein list can be found in Extended Data Table 4-1. ***D***, Log2 fold changes of synaptic proteins within SynGO terms. Downregulated proteins are depicted in blue, upregulated proteins in orange. ***E***, Visual representation of affected STXBP1 interactors identified by string database. Only high confidence interactors, derived from experiments and databases, were included. Affected interactors are depicted in red, unaffected interactors in green and undetected interactors in gray. ***F***, Visual representation of affected STXBP1 interactors identified by co-IP experiments. Co-IP correlation strength to MUNC18-1 is depicted in purple gradient. Undetected interactors are depicted in gray, unaffected interactors in white, and the log2 Fold Change of affected interactors is depicted in blue-orange gradient. ***G***, Overlap of significant proteins at DIV3 and proteins significantly regulated during synaptic silence (24-h TTX treatment) or overactivation (24-h bicuculine treatment; Schanzenbächer et al., 2018). Nine proteins are regulated on TTX treatment as well as on depletion of MUNC18-1, whereas bicuculine treatment shares one protein with *Stxbp1* KO neurons. As for this analysis only first gene names are used, resulting in 15 duplicates, the total number of significant *Stxbp1* KO proteins is lower (384 vs 399 in Fig. 2*C*).

MUNC18-1 is known for its role in SNARE-mediated vesicle fusion in the synapse (Verhage et al., 2000). To better characterize the changes in the synaptic proteome on MUNC18-1 depletion, dysregulated proteins were analyzed in the synaptic gene knowledge base SynGO (Koopmans et al., 2019). A total of 114 dysregulated proteins were annotated as a synaptic protein (Fig. 4*A*), of which 102 were down and 12 upregulated (Extended Data Table 4-1). The vast majority (88%) of the dysregulated synaptic proteins found at DIV2 were also significantly regulated at DIV3, and 63 more synaptic proteins became dysregulated at DIV3. In concordance, 88% of the synaptic proteins were more significant at DIV3 than DIV2 (Fig. 4*B*). A total of 85 of the 114 synaptic proteins were annotated to a biological process (Fig. 4*C*). GO enrichment analysis showed that biological processes in the presynapse were most prominently affected. Affected biological processes showed a high overlap to the functional annotation of MUNC18-1 in the synapse (Fig. 4*C*, left). Indeed, 16 proteins implicated in synaptic vesicle exocytosis, including SNARE partners STX1, SNAP25, and VAMP2, were dysregulated in KO neurons. Fold changes were highly comparable between proteins in different biological processes (Fig. 4*D*). Taken together, *Stxbp1* inactivation dysregulates expression of 114 synaptic proteins, and are strongly related to the known function of *Stxbp1* in the synapse, i.e., presynaptic vesicle exocytosis.

10.1523/ENEURO.0186-22.2022.tab2-1 Extended Data Table 2-1 Protein lists Gene Ontology analysis. Download Table 2-1, XLS file.

10.1523/ENEURO.0186-22.2022.tab3-1 Extended Data Table 3-1 Protein lists overlap other proteomic studies. Download Table 3-1, XLS file.

10.1523/ENEURO.0186-22.2022.tab4-1 Extended Data Table 4-1 Protein list SynGO analysis. Download Table 4-1, XLS file.

10.1523/ENEURO.0186-22.2022.f4-1 Extended Data Figure 4-1 Overlap in significant hits proteomic and transcriptomic analysis. A, Overlap of significant SynGO-annotated transcripts in mass spectrometry analysis. B, Overlap of significant SynGO-annotated proteins in RNAseq analysis. C, Functional annotation of the 36 overlapping genes found both to be regulated at transcript and protein level. Right, All genes significantly dysregulated on protein level in the mass spectrometry analysis. Red highlights overlapping significant transcripts. Download Figure 4-1, EPS file.

Next, we assessed whether MUNC18-1 deficiency affects expression of potential interaction partners. Interactors were identified using the STRING protein-protein interaction database (Jensen et al., 2009) and from previous co-immunoprecipitation (co-IP) experiments (Gonzalez-Lozano et al., 2020). Of the 20 annotated potential interaction partners in the STRING database (>0.9 confidence), nine were detected in our dataset of which six were dysregulated (Fig. 4*E*). Previous co-IP experiments identified 21 interaction partners, of which 11 were detected in our dataset (Fig. 4*F*). Seven were dysregulated. Thus, the majority of detected MUNC18-1 interactions partners are dysregulated in *Stxbp1* KO neurons.

*Stxbp1* KO brains are synaptically silent (Verhage et al., 2000). To investigate whether the effects observed in *Stxbp1* KO neurons are directly caused by a block of synaptic transmission, significant hits were compared with proteomic changes on synaptic silencing by TTX treatment or overactivation by bicuculline (BIC) treatment (Schanzenbächer et al., 2018). A total of 97% of the significant hits on depletion of MUNC18-1 does not overlap with proteomic changes on synaptic silencing or overactivation (Fig. 4*G*; Extended Data Table 3-1). Together, the proteome dysregulation in *Stxbp1* KO neurons shows minimal similarities to proteome changes on altered synaptic activity.

Similar to the proteomic analysis, gene sets related to synaptic transmission were also among the most affected biological processes in *Stxbp1* KO hippocampal transcriptomes. Hence, we next investigated the overlap in synaptic genes between the proteomic and transcriptomic datasets. One third of the SynGO-annotated dysregulated RNA transcripts were detected in the proteomic analysis (Extended Data Fig. 4-1*A*). A total of 40% of these transcripts were also significantly dysregulated on protein level in *Stxbp1* KO neurons. Conversely, nearly all (98%) of the SynGO-annotated significant proteins were detected by RNAseq, and 31% of these were significantly dysregulated on transcript level (Extended Data Fig. 4-1*B*). SynGO functional annotation of the 36 genes that showed dysregulation at both protein and transcript level did not show enrichment of synaptic categories (Extended Data Fig. 4-1*C*). Taken together, limited overlap was observed between dysregulation of synaptic proteins in *Stxbp1* KO neuronal cultures and synaptic transcripts in *Stxbp1* KO hippocampi.

### Loss of MUNC18-1 downregulates proteins implicated in neuron development

**Figure 5.**
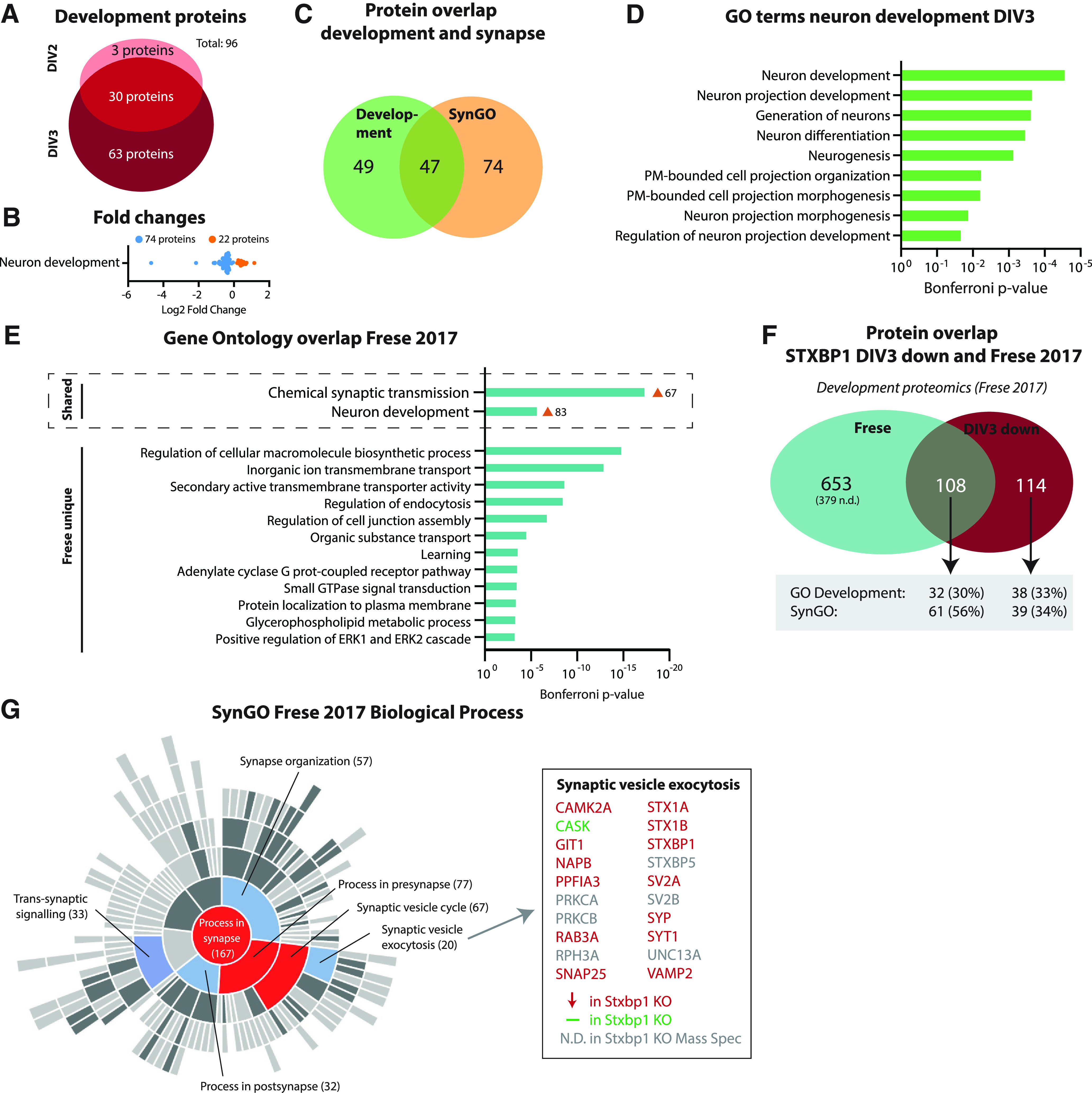
Downregulated proteins in *Stxbp1* KO neurons show high overlap to proteins normally upregulated during this developmental stage. ***A***, A total of 96 proteins annotated to development are dysregulated in *Stxbp1* KO neurons. Three developmental proteins were only significant at DIV2, 30 developmental proteins significant at DIV2 and DIV3, and 63 development proteins only significant at DIV3. ***B***, Log2 fold changes of proteins associated with development. Downregulated proteins are depicted in blue, upregulated proteins in orange. ***C***, Overlap in proteins annotated in SynGO and GO development. A total of 49 proteins were only annotated to development, 47 proteins to both development and SynGO, and 74 proteins were only annotated in SynGO. ***D***, All individual GO terms that were included in the neuron development cluster. Shown are the Bonferroni correct *p*-values. ***E***, GO enrichment analysis of significant upregulated proteins between DIV1 and DIV5 (Frese et al., 2017). Shown are the Bonferroni correct *p*-values. GO term clusters chemical synaptic transmission and neuron development were shared with *Stxbp1* KO neurons. ***F***, Overlap of significant proteins at DIV3 and proteins significantly upregulated between DIV1 and DIV5 (i.e., Frese dataset). A total of 653 proteins were unique for the Frese dataset, 108 proteins shared between the two datasets, and 114 unique for *Stxbp1* KO neurons. Of the 108 proteins shared, 32 were annotated to GO development and 61 in SynGO. As for this analysis, only first gene names are used, resulting in 15 duplicates, the total number of significant *Stxbp1* KO proteins is lower (384 vs 399 in Fig. 2*C*). ***G***, 167 proteins in the Frese dataset were categorized in SynGO biological processes. Shown is a sunburst plot with color-coded enrichment significance of every GO term. Right are depicted all proteins in the Frese dataset associated with synaptic vesicle exocytosis, color-coded to dysregulation in *Stxbp1* KO neurons. Significantly regulated transcription factors are shown in Extended Data Figure 5-1.

To further understand the dysregulation of neuron development in *Stxbp1* KO neurons, we studied this biological process in more detail. *Stxbp1* KO neurons showed significantly altered expression levels of 96 proteins involved in neuron development (Fig. 5*A*). Thirty proteins were dysregulated at both time points, while 63 were specific for DIV3. The vast majority (77%) were downregulated compared with control neurons (Fig. 5*B*), typically between 0 and 1 fold change (log2), except two proteins (MUNC18-1 and syntaxin-1) that were drastically changed. Of these, 49% are SynGO-annotated (synaptic) proteins (Fig. 5*C*). Within the GO cluster term neuron development, nine GO terms were significantly enriched (Fig. 5*D*). Five terms contained the word “projection,” which concerns a specific aspect of neuron development. Together, *Stxbp1* inactivation results in downregulation of proteins implicated in neuron development, which extends beyond regulation of synaptic proteins only. Within the general biological process of neuron development, especially processes related to development of (neurite) projections were affected.

10.1523/ENEURO.0186-22.2022.f5-1 Extended Data Figure 5-1 Regulated transcription factors in Stxbp1 KO neurons. A, Among the 399 significantly dysregulated proteins at DIV3, seven were annotated as TF (Lambert et al., 2018). Five TFs (underscore) are involved in neuron development. Download Figure 5-1, EPS file.

The downregulation of this group of proteins in *Stxbp1* KO neurons could indicate a halt in development. Hence, we compared the proteins downregulated in *Stxbp1* KO neurons to a set of proteins typically upregulated in neuronal development between DIV1 and DIV5 (Frese et al., 2017). Comparable to *Stxbp1* KO neurons, GO analysis of this dataset revealed enrichment of the biological processes chemical synaptic transmission and neuron development (Fig. 5*E*); 49% of the downregulated proteins in *Stxbp1* KO neurons were significantly different between DIV1 and DIV5 (Fig. 5*F*; Extended Data Table 3-1). Of these, 56% were annotated in the SynGO database, and 30% were GO annotated under neuron development. To further understand the overlap in synaptic proteins, the Frese dataset was annotated in the SynGO database (Fig. 5*G*). Enriched synaptic biological processes were highly comparable to *Stxbp1* KO neurons (compare Fig. 5*G* and 4*C*), with an enrichment in presynaptic proteins involved in synaptic vesicle exocytosis. Thirteen of the 14 detected proteins within this biological process were downregulated in *Stxbp1* KO neurons. Taken together, the downregulation of developmental and synaptic proteins in *Stxbp1* KO neurons shows high overlap to proteins normally upregulated at this developmental stage.

Neuronal development requires the upregulation of neurogenic transcription factors (TFs) and downregulation of anti-neuronal TFs, activating genetic programs important for further neuron differentiation (Vieira et al., 2018). To investigate whether loss of MUNC18-1 affects TF expression levels, we examined whether TFs were among the significant dysregulated proteins (Lambert et al., 2018). The proteomic screen detected a total of 61 TFs, of which seven were dysregulated in *Stxbp1* KO neurons (Extended Data Fig. 5-1). Five of these seven TFs, including CREB1, are shown to be involved in critical processes during neuron development (Rowlands et al., 1994; Lonze and Ginty, 2002; Rottkamp et al., 2008; Hutnick et al., 2009; Cohen et al., 2017). Together, loss of MUNC18-1 results in altered expression levels of several TFs implicated in neuronal development.

## Discussion

MUNC18-1 plays an essential role in neuronal viability during development, independent of its known function in SNARE-mediated vesicle fusion. In the present study, we examined transcriptome and proteome changes on depletion of MUNC18-1 during the critical time window preceding neuronal cell death. We show that loss of MUNC18-1 results in extensive remodeling of transcriptomic and proteomic profiles, and particularly affects proteins related to synaptic transmission and neuron development.

The extensive downregulation of proteins implicated in synaptic function and development suggests an important role for MUNC18-1 in early neuron development. Neurons undergo multiple well-coordinated stages of development after early neurogenesis, which has been extensively studied in neurons *in vitro* (Dotti et al., 1988; Frese et al., 2017). First, neurons form lamellipodia (stage 1; DIV0) that develop into short neurites (stage 2; DIV1). One of the neurites develops into an axon (stage 3; DIV2–DIV3), whereas the others become dendrites (stage 4; DIV4). From this point, neurons already upregulate expression of synaptic proteins (Frese et al., 2017), but it is not until DIV7 that neurons start to form synapses (stage 5). Previous studies in brain slices and neurons *in vitro* have shown that initial neurogenesis and brain organization do not depend on MUNC18-1/STXBP1 (Verhage et al., 2000; Bouwman et al., 2004; Santos et al., 2017). Furthermore, *Stxbp1* KO neurons initiate outgrowth of neurites which polarize into axons and dendrites (Heeroma et al., 2004; Broeke et al., 2010; Santos et al., 2017). However, from thereon development abates. KO neurons show reduced speed of outgrowth at DIV3, resulting in lower total neurite length which continues to exacerbate at DIV4 (Broeke et al., 2010). Concordantly, neurite development was most prominent among the developmental processes affected at DIV2 and DIV3 in this study. In addition, a high overlap was observed between the synaptic proteins downregulated in absence of MUNC18-1 and synaptic proteins normally upregulated at this developmental stage (Frese et al., 2017). Together, it is conceivable that the absence of MUNC18-1 results in developmental delay between the stages of neurite outgrowth and early synaptogenesis.

Concurrent with a delay in neuron development, *Stxbp1* KO neurons *in vitro* die at DIV3–DIV4 (Santos et al., 2017). Interestingly, the proteome profiling in this study did not identify known cell death-related processes. Different and not currently annotated cell death pathways might operate in *Stxbp1* KO neurons. Moreover, it is plausible that the degeneration occurs very fast, precluding the detection of cell death-related proteins in the total neuron population where every neuron dies at a slightly different time point. Indeed, live-cell imaging of *Stxbp1* KO neurons showed that cell death occurred within hours after initial onset (F. Feringa, unpublished observation). Considering this rapid cell death together with the observed arrest in development, MUNC18-1 might serve as a checkpoint during development. To continue to the next developmental phase, the presence of MUNC18-1 might be required, otherwise neurons are developmentally arrested and redirected into cell death. Such cellular checkpoints as quality control have been described in other processes (Barnum and O’Connell, 2014; Sancho and Ouchi, 2015). For instance, proliferating cells have incorporated a DNA damage checkpoint (Dasika et al., 2000). When this checkpoint is activated, the cell cycle is arrested to allow repairing the damaged DNA. If the cell is not capable in repairing the DNA, programmed apoptotic cell death is initiated. In neurons, it has been proposed that several developmental stages require functional feedback to move to the next stage (Ben-Ari and Spitzer, 2010). Neurons failing to provide such feedback are developmentally arrested. For instance, neuronal migration requires expression of doublecortin (DCX). In absence of DCX, migration is hindered and neurons remain immature (Ackman et al., 2009). Similar feedback loops have been described for axon guidance and neuronal specification (Ben-Ari and Spitzer, 2010).

The nature of such a MUNC18-1-dependent checkpoint mechanism between the stage of neurite outgrowth and early synaptogenesis remains elusive. It is unlikely that a lack of synaptic activity per se underlies the developmental arrest. Proteome changes in *Stxbp1* KO neurons show minimal overlap with proteins normally regulated during loss or overactivation of synaptic activity (Fig. 4*G*). Moreover, in other models of synaptic silence, for instance, in MUNC13-1/2 DKO or TeNT-treated neurons, the absence of synaptic transmission does not profoundly affect levels of synaptic proteins, synaptic morphogenesis or neuron development (Varoqueaux et al., 2002; Sando et al., 2017). Indeed, it has been shown that neurite outgrowth, polarization and initial synapse formation all develop independent from activity, while further synapse specification is thought to be activity-dependent (Südhof, 2018). Moreover, MUNC18-1’s critical role in dense-core vesicle exocytosis is not sufficient to explain a checkpoint function, as models that (specifically) block dense-core vesicle release do not show indications for developmental arrest and/or neurodegeneration (Persoon et al., 2019; Hoogstraaten et al., 2020). Hence, synaptic activity and dense-core vesicle release are not critical checkpoints in neurons, excluding this as underlying checkpoint of MUNC18-1 dependency.

Multiple alternative mechanisms are plausible. For instance, it is possible that MUNC18-1 directly functions as a transcriptional regulator or translational modifier. It has been reported previously that MUNC18-1 localizes to the nucleus and binds DNA (V. M. Sharma et al., 2005), although this has not been confirmed by other studies. The structure of MUNC18-1 does not contain established DNA or RNA binding domains. Hence, evidence for a direct role of MUNC18-1 in transcription/translation is limited. Alternatively, MUNC18-1-dependent regulation may start at the protein level. For instance, it has already been demonstrated that in absence of MUNC18-1, syntaxin-1 is trapped in the Golgi (Rowe et al., 1999; Arunachalam et al., 2008). Although *Stxbp1* KO neurons do not show general impairments in Golgi exit (van Berkel et al., 2021), it is plausible that specific synaptic proteins are also trapped in the Golgi and remain mislocalized. This could initiate a cascade of events involving feedback loops on either the gene or RNA level to larger sets of proteins ultimately resulting in developmental arrest. Alternatively, MUNC18-1 might directly control gene-programs during development, for instance, by affecting levels of key developmental TFs (Extended Data Fig. 5-1).

It is currently unknown whether the proposed function of MUNC18-1 as a checkpoint gene in neuron development is shared with other proteins like its SNARE partners. Neurons also critically depend on Syntaxin-1 and SNAP25 for viability during early development (Delgado-Martínez et al., 2007; Peng et al., 2013; Vardar et al., 2016; Santos et al., 2017). Although mass spectrometry analysis has not yet been performed in both KO models, expression analysis of a selected set of proteins indicates that depletion of Syntaxin-1 results in downregulation of synaptic proteins similar as MUNC18-1 depletion (Vardar et al., 2016). In SNAP25 KO neurons, protein dysregulation is less apparent (Washbourne et al., 2002; Arora et al., 2017). For other proteins involved in SNARE-mediated fusion, such as VAMP2, MUNC13, RIM, and SYT1, no evidence is found for critical roles in regulating expression levels of synaptic and developmental proteins, and their absence does not trigger neuronal degeneration (Geppert et al., 1994; Schoch, 2001; Varoqueaux et al., 2002; Kaeser et al., 2011). Hence, MUNC18-1’s role as a checkpoint gene of synapse development is presumably shared with Syntaxin-1, and potentially SNAP25, but not with other key factors of the secretion machinery.

Inactivation of several other synaptic genes such as MUNC13-1 + 2, CAPS-1 + 2, Complexin-1 + 2 or CSPα leads to lethality *in vivo*, yet primary neuronal cultures survive (Reim et al., 2001; Varoqueaux et al., 2002; Fernández-Chacón et al., 2004; Jockusch et al., 2007; M. Sharma et al., 2012). None of these proteins is required for synapse formation. Conversely, inactivation for genes important for the initial steps of synapse formation, such as cell adhesion molecules (CAMs), does not lead to reduced neuronal survival *in vitro* or profound effects on neuron development (Südhof, 2018). Interestingly, the effects of single CAM KOs on synapse formation are relatively mild, potentially because of redundancy (Robbins et al., 2010; Yim et al., 2013; Südhof, 2018). Indeed, stronger effects are often observed when multiple CAM proteins are depleted (Ko et al., 2011; L. Y. Chen et al., 2017). Thus, the essential role of MUNC18-1, and potentially Syntaxin-1 and SNAP25, during neuron development and synapse formation is exceptional among synaptic proteins.

Heterozygous loss-of-function variants in *STXBP1* are among the most prevalent found in neurodevelopmental disorders (López-Rivera et al., 2020). Developmental delay is a shared feature of all *STXBP1* variant carries (Stamberger et al., 2016; Abramov et al., 2021; Xian et al., 2022). Hence, the uncovered role of MUNC18-1 in regulating (synaptic) development could potentially play a role in the pathobiology of these patients. To date, no evidence is found that heterozygous levels of MUNC18-1 majorly affect neuronal viability or brain development *in vivo* and *in vitro* (Verhage et al., 2000; Toonen et al., 2006; Kovacevic et al., 2018; W. Chen et al., 2020). In patients, no clear signs of neurodegeneration or brain structural abnormalities are observed (Di Meglio et al., 2015; Stamberger et al., 2016; Abramov et al., 2021) Thus, it is unlikely that patients’ neurons fail to pass the proposed checkpoint during development. It is, however, possible that heterozygous MUNC18-1 levels already have an effect on the regulation of (synaptic) development, producing a milder phenotype than KO neurons but still contribute to the pathobiology in patients.

## Synthesis

Reviewing Editor: Matthew Grubb, King’s College London Decisions are customarily a result of the Reviewing Editor and the peer reviewers coming together and discussing their recommendations until a consensus is reached. When revisions are invited, a fact-based synthesis statement explaining their decision and outlining what is needed to prepare a revision will be listed below. The following reviewer(s) agreed to reveal their identity: Shuzo Sugita. Note: If this manuscript was transferred from JNeurosci and a decision was made to accept the manuscript without peer review, a brief statement to this effect will instead be what is listed below.

Reviewer 1:

The deletion of MUNC18-1 protein causes severe neurodegeneration in mice (Verhage et al., 2002), yet the underlying mechanisms are unknown. While many efforts have been made to resolve the enigma, the present work, which includes RNAseq and proteomics, would be arguably the most extensive effort. While the results did not underpin the mechanisms, they at least seem to exclude the potential mechanism of the cell death pathway activation (Fig. 3). Therefore, I would think that the present work contains important information for other scientists in the field.

Minor points:

1) In the abstract, the authors state: “Absence of presynaptic protein MUNC18-1 (gene: Stxbp1) leads to neuronal cell death at an immature stage before synapse formation, independent from its known essential function in synaptic vesicle exocytosis.” I would suggest that the authors remove “independent from its known essential function in synaptic vesicle exocytosis.”

I understand that the authors want to say that the lack of exocytosis per se by MUNC18-1 deletion is not causing neuronal cell death. Yet, the authors proteomics data showed that MUNC18-1 deletion leads to downregulation of 114 synaptic proteins enriched in presynaptic vesicle release (Fig. 4). Therefore, many readers would be confused by the sentence of “independent from its known essential function in synaptic vesicle exocytosis” in abstract.

2) I understand that neurodegeneration in the absence of MUNC18-1 starts at E13 (Verhage et al., 2002). The authors used E18 hippocampal tissue of Munc18-1 KO mice as the samples shortly before the moment of cell death. However, I am concerned whether the E18 hippocampus may already suffer from neurodegeneration. I strongly suggest that the authors show the photograph of hippocampal tissues from Munc18-1 KO mice with that from WT mice side by side in Fig. 1. Ideally, if possible, the authors should demonstrate that the E18 Munc18-1 KO hippocampal tissues do not suffer from neurodegeneration by immunohistochemistry.

Reviewer 2:

Munc18-a/Stxbp1 is an essential protein involved in synaptic vesicle exocytosis and neurotransmitter release. Interestingly, Verhage et al also show in a seminal article that loss of Munc18-a/Stxbp1 leads to neuronal cell death, the mechanism of which is still not elucidated.

Here Verhage and coworkers used transcriptomics, proteomics and bioinformatics to analyse defects in the brain of Munc18-a/Stxbp1 KO brains and cultured neurons.

Using state-of-the-art methods, they convincingly show that Munc18-a/Stxbp1 KO hippocampi have defects in expressing genes related to synapse, immunity, lipids and actin. Corresponding cultured neurons until DIV3 and DIV5 show primary defects in proteins related to synapse and neuronal development. An in-depth analysis of the data did not uncover any apparent link to cell death-related genes but rather pointed to the downregulation of proteins involved in synaptic vesicle release, a fraction being Munc18-a/Stxbp1 interactions such as Stx1A. Another analysis showed that downregulated proteins in Munc18-a/Stxbp1 neurons are normally upregulated during the developmental stage corresponding to DIV3. Comparison with published data on silenced neuronal cultures treated by TTX shows apparent differences with the present dataset.

The authors conclude with the role of Munc18-a/Stxbp1 as a neuronal checkpoint in addition to its critical role in synaptic vesicle exocytosis. They raise the interesting hypothesis that Munc18-a/Stxbp1 could regulate the transcription of genes and have a function in the nucleus.

In spite of not providing a molecular and cellular mechanism, this article is based on solid data and a very thoughtful discussion. It is extremely interesting for the synapse community.

The authors did not comment on the role of Munc18-a/Stxbp1 in large dense-core vesicle exocytosis. This reviewer thinks the authors should discuss whether or not a defect in the release of neuropeptides could participate in the proposed checkpoint role of Munc18-a/Stxbp1. It would also be interesting to briefly comment on how these new data could shed light on the STXBP1-related disorders in humans.

## Author Response

We thank the reviewers for the insightful and constructive comments. We have revised the manuscript to address their comments.

Please find below a point-by-point reply to the reviewers with the reviewer’s text in blue and our answers in black.

Reviewer 1:

The deletion of MUNC18-1 protein causes severe neurodegeneration in mice (Verhage et al., 2002), yet the underlying mechanisms are unknown. While many efforts have been made to resolve the enigma, the present work, which includes RNAseq and proteomics, would be arguably the most extensive effort. While the results did not underpin the mechanisms, they at least seem to exclude the potential mechanism of the cell death pathway activation (Fig. 3). Therefore, I would think that the present work contains important information for other scientists in the field.

We thank the reviewer for his/her positive evaluation of the manuscript.

Minor points:

1) In the abstract, the authors state: “Absence of presynaptic protein MUNC18-1 (gene: Stxbp1) leads to neuronal cell death at an immature stage before synapse formation, independent from its known essential function in synaptic vesicle exocytosis.” I would suggest that the authors remove “independent from its known essential function in synaptic vesicle exocytosis.”

I understand that the authors want to say that the lack of exocytosis per se by MUNC18-1 deletion is not causing neuronal cell death. Yet, the authors proteomics data showed that MUNC18-1 deletion leads to downregulation of 114 synaptic proteins enriched in presynaptic vesicle release (Fig. 4). Therefore, many readers would be confused by the sentence of “independent from its known essential function in synaptic vesicle exocytosis” in abstract.

We agree with the reviewer and have removed the sentence from the abstract (line 3).

2) I understand that neurodegeneration in the absence of MUNC18-1 starts at E13 (Verhage et al., 2002). The authors used E18 hippocampal tissue of Munc18-1 KO mice as the samples shortly before the moment of cell death. However, I am concerned whether the E18 hippocampus may already suffer from neurodegeneration. I strongly suggest that the authors show the photograph of hippocampal tissues from Munc18-1 KO mice with that from WT mice side by side in Fig. 1. Ideally, if possible, the authors should demonstrate that the E18 Munc18-1 KO hippocampal tissues do not suffer from neurodegeneration by immunohistochemistry.

This is a valid point. We have now performed immunohistochemistry using nuclear marker DAPI and apoptosis marker cleaved caspase 3 on E18 hippocampal tissue from WT and Stxbp1 KO mice. The sections show that, in contrast to lower brain areas, the hippocampus of Stxbp1 KO mice is still intact with comparable cell number and density. A minority of cells show increased reactivity against cleaved-caspase 3, indicating that the first cells start to degenerate in the hippocampus. We have included a typical example of these sections as extended Figure 1-1 and referred to in text (lines 64-66).

Reviewer 2:

Munc18-a/Stxbp1 is an essential protein involved in synaptic vesicle exocytosis and neurotransmitter release. Interestingly, Verhage et al also show in a seminal article that loss of Munc18-a/Stxbp1 leads to neuronal cell death, the mechanism of which is still not elucidated.Here, Verhage and coworkers used transcriptomics, proteomics and bioinformatics to analyse defects in the brain of Munc18-a/Stxbp1 KO brains and cultured neurons. Using state-of-the-art methods, they convincingly show that Munc18-a/Stxbp1 KO hippocampi have defects in expressing genes related to synapse, immunity, lipids and actin. Corresponding cultured neurons until DIV3 and DIV5 show primary defects in proteins related to synapse and neuronal development. An in-depth analysis of the data did not uncover any apparent link to cell death-related genes but rather pointed to the downregulation of proteins involved in synaptic vesicle release, a fraction being Munc18-a/Stxbp1 interactions such as Stx1A. Another analysis showed that downregulated proteins in Munc18-a/Stxbp1 neurons are normally upregulated during the developmental stage corresponding to DIV3. Comparison with published data on silenced neuronal cultures treated by TTX shows apparent differences with the present dataset.

The authors conclude with the role of Munc18-a/Stxbp1 as a neuronal checkpoint in addition to its critical role in synaptic vesicle exocytosis. They raise the interesting hypothesis that Munc18-a/Stxbp1 could regulate the transcription of genes and have a function in the nucleus. In spite of not providing a molecular and cellular mechanism, this article is based on solid data and a very thoughtful discussion. It is extremely interesting for the synapse community.

We thank the reviewer for his/her positive evaluation of the manuscript.

The authors did not comment on the role of Munc18-a/Stxbp1 in large dense-core vesicle exocytosis. This reviewer thinks the authors should discuss whether or not a defect in the release of neuropeptides could participate in the proposed checkpoint role of Munc18-a/Stxbp1.

We agree that MUNC18-1’s essential role in the release of dense-core vesicles could provide an interesting explanation on how MUNC18-1 regulates (synapse) development. However, in models where dense-core vesicle release is completely abolished (e.g., Rab3 KO or Tetanus administration to cleave VAMPs), no signs of developmental arrest and/or neurodegeneration are observed (Persoon et al., 2019, Hoogstraaten et al., 2020). Hence, it is unlikely that the block of dense-core vesicle release underlies MUNC18-1’s role in (synapse) development. We have included MUNC18-1’s role in dense-core exocytosis, and whether this could explain the proposed checkpoint role, in the introduction (lines 29-32) and discussion (lines 291-294).

It would also be interesting to briefly comment on how these new data could shed light on the STXBP1-related disorders in humans.

We share the opinion of the reviewer that the uncovered role of MUNC18-1 in developmental regulation could be implicated in the pathogenesis of STXBP1-related disorders. In addition to the significance statement, we have now included a section on this in the discussion (lines 331-341).
