# Supplementary material for: Dysregulation of Synaptic and Developmental Transcriptomic/Proteomic Profiles upon Depletion of MUNC18-1
Source: eNeuro. 2022 Nov 9;9(6):ENEURO.0186-22.2022. doi: 10.1523/ENEURO.0186-22.2022 (PMC9668351; doi:10.1523/ENEURO.0186-22.2022)
Supplement: Extended Data Table 1-1 — qPCR primers. Download Table 1-1, DOCX file. [file enu-eN-NWR-0186-22-s11.docx]

| Primer | Sequence |
| --- | --- |
| Stxbp1-1F | GTGGACCAGTTAAGCATGAGG |
| Stxbp1-1R | GCTCTCGGCGCTTGTTGAT |
| Stx1a-1F | AAGATTGCCGAAAACGTGGAG |
| Stx1a-1R | TGCTCAATGCTCTTTAGCTTGG |
| Doc2a-1F | ATCGCATGACCATCAACATCC |
| Doc2a-1R | GCGAGGGAAGTAGTCGGAGA |
| Dnm1-2F | AATATGCCGAGTTCCTGCACT |
| Dnm1-2R | GTCTCAGCCTCGATCTCCAG |
| Mmp12-1F | GGGCTGCTCCCATGAATGAC |
| Mmp12-1R | CCAGAGTTGAGTTGTCCAGTTG |
| Slc6a7-1F | ACCTGGATGTAGACTTCGCAG |
| Slc6a7-1R | CGCCAGACATTTCCCAAGC |
| Snap25-F | CAACTGGAACGCATTGAGGAA |
| Snap25-R | GGCCACTACTCCATCCTGATTAT |
| Col5a1-F | CTTCGCCGCTACTCCTGTTC |
| Col5a1-R | CCCTGAGGGCAAATTGTGAAAA |
| Ddn-F | GACCCTGGGGACTAAGCGA |
| Ddn-R | ACATCCCGGTAGATTCGAGGA |
